# The Development of Microscopic Imaging Technology and its Application in Micro- and Nanotechnology

**DOI:** 10.3389/fchem.2022.931169

**Published:** 2022-07-05

**Authors:** Yong Wang, Xiushuo Zhang, Jing Xu, Xiangyu Sun, Xiaolong Zhao, Hongsheng Li, Yanping Liu, Jingjing Tian, Xiaorui Hao, Xiaofei Kong, Zhiwei Wang, Jie Yang, Yuqing Su

**Affiliations:** ^1^ Laboratory of Optical Detection and Imaging, School of Science, Qingdao University of Technology, Qingdao, China; ^2^ Quantum Physics Laboratory, School of Science, Qingdao University of Technology, Qingdao, China; ^3^ Qingdao Technology Innovation Center of Remote Sensing and Precise Measurement, Qingdao, China; ^4^ Torch High Technology Industry Development Center, Ministry of Science and Technology, Beijing, China

**Keywords:** microscopic, imaging, micro-nano, review, expectation

## Abstract

As a typical microscopic imaging technology, the emergence of the microscope has accelerated the pace of human exploration of the micro world. With the development of science and technology, microscopes have developed from the optical microscopes at the time of their invention to electron microscopes and even atomic force microscopes. The resolution has steadily improved, allowing humans to expand the field of research from the initial animal and plant tissues to microorganisms such as bacteria, and even down to the nanolevel. The microscope is now widely used in life science, material science, geological research, and other fields. It can be said that the development of microscopes also promotes the development of micro- and nanotechnology. It is foreseeable that microscopes will play a significant part in the exploration of the microworld for a long time to come. The development of microscope technology is the focus of this study, which summarized the properties of numerous microscopes and discussed their applications in micro and nanotechnology. At the same time, the application of microscopic imaging technology in micro- and nanofields was investigated based on the properties of various microscopes.

## 1 Introduction

A microscope is an instrument that magnifies the image of an observed object. It has a history of 400 years since its invention at the end of the 16th century. Because of its ability to magnify images, the microscope has played a vital part in the investigation of the microscopic world. Microscopic imaging technology evolves in tandem with human awareness of the microcosmic world. For the time being, the microscope includes the following three types according to the principles: optical microscope, electron microscope, and scanning probe microscope. The different principles make the use and contribution significantly different. In this study, we will discuss the evolution of the microscope and its applications in micro- and nanotechnology.

## 2 The Optical Microscope

### 2.1 Introduction to Optical Microscope

The optical microscope is the first human-made microscope. It has played an extremely important role in the initial stage of human understanding of the micro world. For the past 400 years, the basic construction of the optical microscope remained stable without major changes, including objective lens, eyepiece, light source, condenser, and the mechanical structure. The resolution of traditional optical microscopes meets the Abbe formula: $\delta =\frac{0.61\lambda }{n\sin\alpha }$ ,

where *δ* is the optical microscope resolution, *λ* is the wavelength of light, n is the refractive index, and *α* is the aperture angle. The resolution limit of an optical microscope calculated is about 0.2 microns, so an optical microscope is widely used at a micron level, especially for the study of cells in biology and medicine. However, further observations of smaller objects are limited.

**FIGURE 1:**
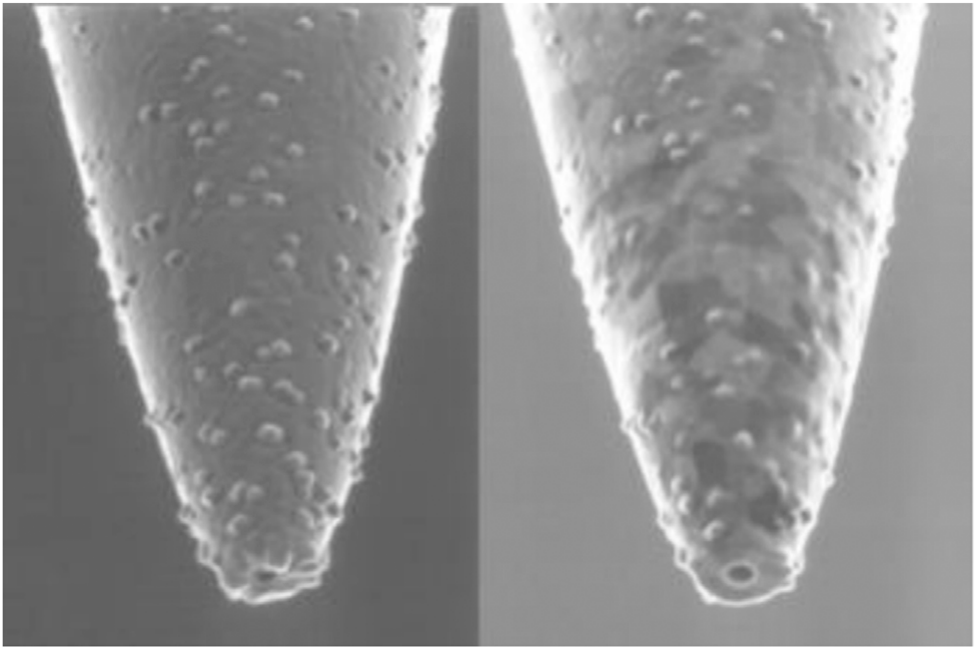
Near-field scanning optical microscope probe (Dunn et al., 2009).

With the development of science and technology, near-field optical microscopy (NSOM) has been developed in recent years. Compared to the traditional optical microscope, its resolution has substantially enhanced. The structure includes the local light source, laser, fiber probe sample station, and optical amplification system. The characteristic of the near-field microscope is that at least one illumination and imaging must work in the near field, while both traditional optical microscopes work in the far field. The resolution of traditional far-field optical microscopes has been limited to wavelength *λ* or aperture n⁡sin⁡α . The working mode of the near-field optical microscope is to obtain information on fine structure and fluctuation smaller than the super-resolution limit of wavelength from the electromagnetic field (evanescent field) in the near field, and then transform the evanescent field containing this information into a propagation field that can transmit energy, allowing the detector and imaging device placed in the distance to receive the information in the evanescent field. It works by the reversibility of light, which means that when the direction of light is reversed in diffraction, the light will go backward along the incident route. Therefore, when the evanescent wave containing super-resolution information is used to irradiate objects with fine structure or spatial fluctuation smaller than the wavelength, such as gratings and small holes, these gratings or small holes can convert the evanescent wave into a propagation wave containing super-resolution information, which can be accepted by distant detectors. Currently, the near-field scanning optical microscope is used extensively in biomedical research. The probe section of a near-field scanning optical microscope is shown in Figure 1 cited from JP Dunn’s article in 2014.

**FIGURE 2:**
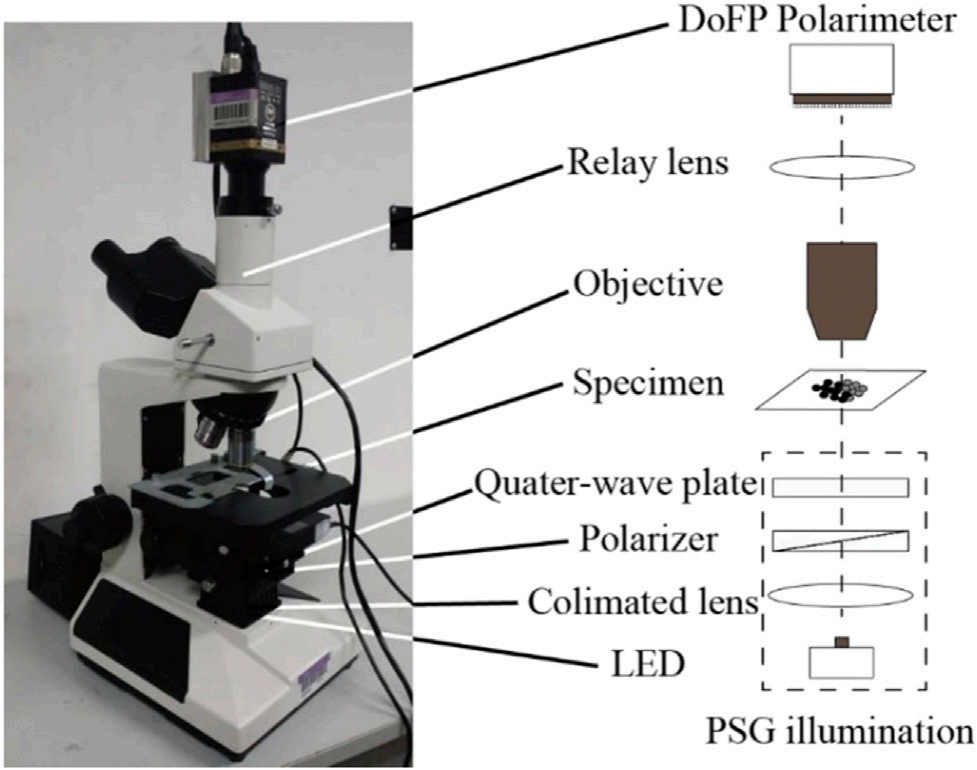
Polarization microscope based on division-of-focal plane (DoFP) polarimeter (Chang et al., 2016).

Increasing the information dimension is one of the important directions to improving the traditional optical microscope. Polarization is an inherent property of light, which is more sensitive to microstructure. Through polarization measurement, we can obtain more sample information including light intensity (Wang et al., 2018; Wang et al., 2020). Therefore, realizing polarization measurement in a traditional microscope has unique advantages in detecting samples. This is the polarization microscope. The main structure of a polarization microscope is similar to that of a traditional optical microscope, but polarizers and analyzers are added into its optical path system to realize polarization measurement. The picture from J Chang’s research article in 2016 is quoted in Figure 2 to show the polarization microscope.

**FIGURE 3:**
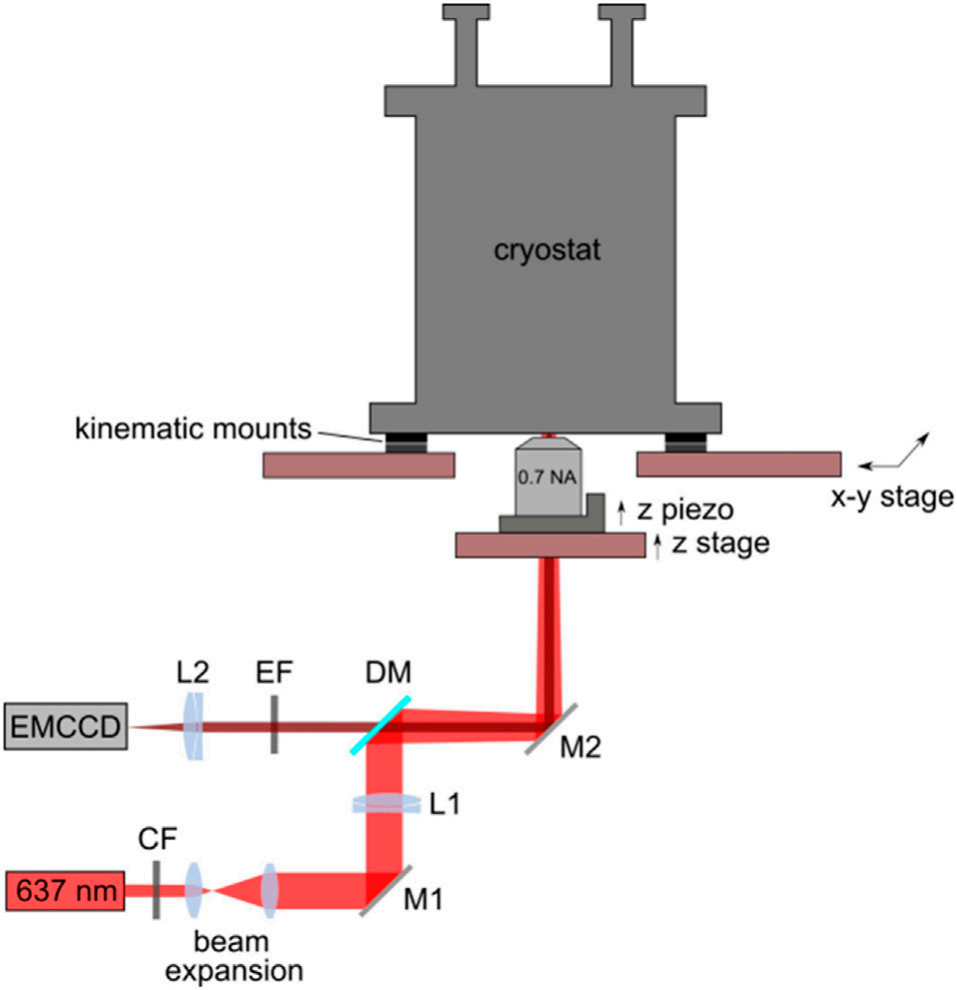
Structure diagram of low temperature fluorescence microscope (Li et al., 2015).

At the same time, it should be noted that a fluorescence microscope is another traditional optical principle-based microscope. Many materials fluoresce when exposed to a specifically engineered excitation light source, which is often ultraviolet, blue violet, or green light. Other non-fluorescent materials can be stained, and associated materials can then be monitored using UV light. Weixing Li et al. (2015) are cited in this study to demonstrate the structural properties of a low-temperature fluorescence microscope as shown in Figure 3.

It is worth noting that in recent years, with the advent of super-resolution fluorescence microscopy, fluorescence microscopy can get rid of the resolution of traditional optical microscopy. Photoactivated localization microscopy (PALM), stochastic optical reconstruction microscopy (STORM), and stimulated emission depletion (STED) are currently used to improve microscopic imaging in the direction perpendicular to light propagation, while STED-4 3D imaging and 3D SSIM imaging saturated structure illumination (SSIM) are used to improve microscopic imaging in the direction of light propagation. The resolution is at the nanoscale. It is widely used in the field of life science to observe the expression and embodiment of biological macromolecules and cellular functions in living cells at the molecular level.

**FIGURE 4:**
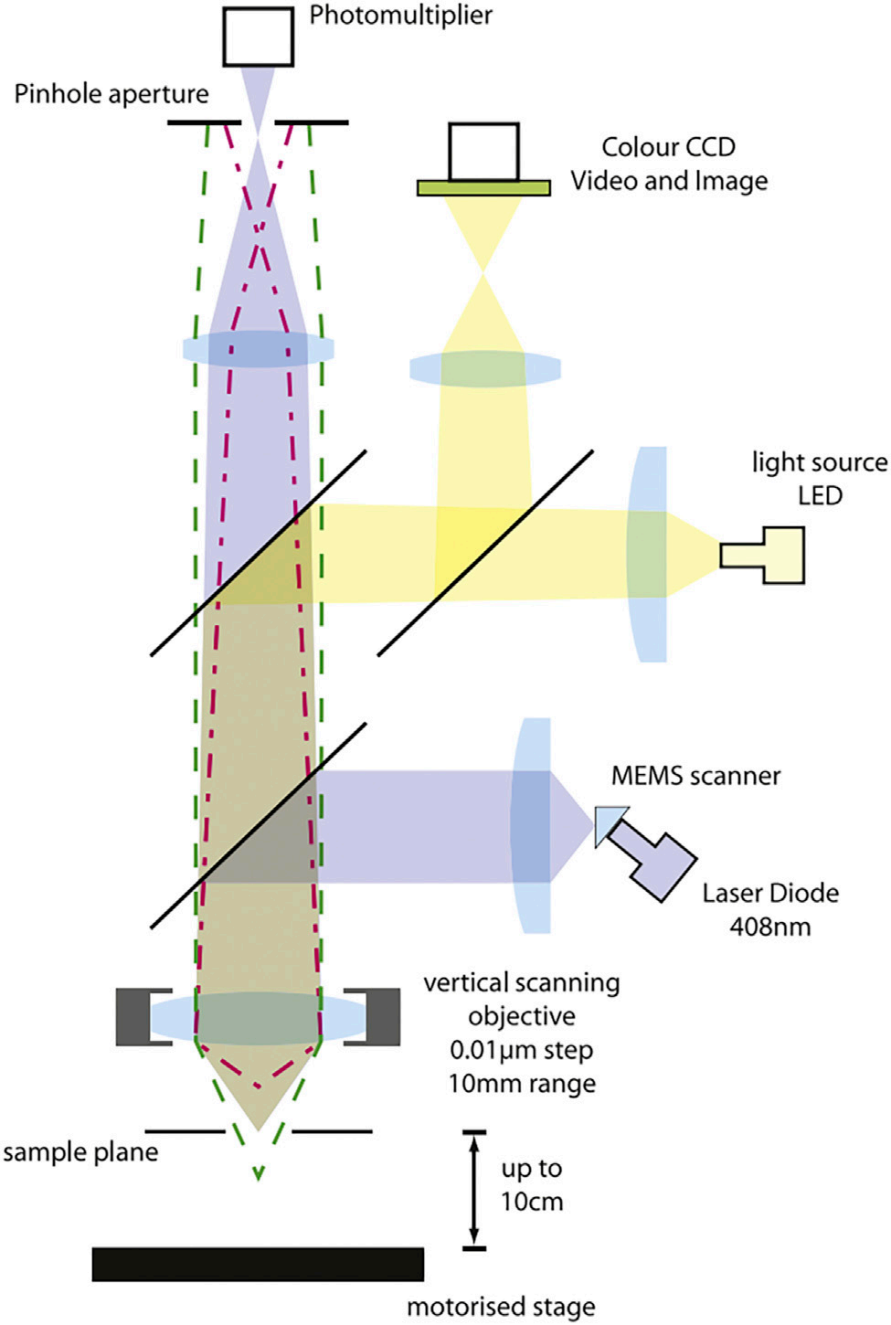
Principle diagram of laser confocal scanning microscope (Evans and Donahue, 2008).

It must be mentioned that laser scanning confocal microscopy has become one of the important microscopy tools nowadays. A laser scanning confocal microscope is a set of observation, analysis, and output systems. The key technology is conjugate focusing based on a traditional optical microscope. It consists primarily of a laser light source, an automatic microscope, a scanning module (which contains a confocal optical route channel and pinhole, a scanning mirror, and a detector), a digital signal processor, a computer, and also an image output device, among others. In Figure 4, there is an image from a research published by AA Evans in 2008 to show the structure and principle of laser scanning confocal microscopy.

### 2.2 Application of Optical Microscope in Micro- and Nanotechnology

#### 2.2.1 Application of Traditional Optical Microscope

The imaging process in optical microscopy is nondestructive to the material. It is a reasonably simple observation technique, which can realize real-time imaging. The contemporary conventional optical microscope, however, has fewer applications at the nanoscale scale than other microscopes because its resolution is limited by light diffraction, and its applications are mostly centered on the micron size. Traditional optical microscopy is still one of the most important detection tools in the field of life sciences and clinical medicine because of the advantages of non-destructive observation of samples, real-time imaging, and a relatively simple observation process. In a study published in 2015, Fu Rong et al. used urine routine combined with optical microscopy for morphological examination of urine erythrocytes and thus for early diagnosis of Alport syndrome (Rong et al., 2015). R Hauser et al. investigated the effect of optical and tracking features on the accuracy of an optical microscope guidance system used in sinus surgery in 1999 (Hauser and Westermann, 1999). In addition, Kamimura, Shinji et al., in their study in 1987, proposed a method of using a prism to divide the pinhole magnified image of optical microscope into two parts and directly measuring the nano displacement by measuring the light intensity difference (Kamimura, 1987). At the same time, as one of the branches of optical microscope, metallographic microscope also plays an important role in the detection of metal and rock structure. The findings of a study conducted by B Szala et al. (2013) using metallographic microscopy for the observation of corroded cross-sectional layers revealed that in addition to reacting to the color and thickness of the corroded layer, metallographic microscopy can also try to determine the crystal structure generated, while the location of inclusions, cracks, and blisters in the sample will help to determine the history of the sample (Szala et al., 2013).

#### 2.2.2 Application of Near-Field Scanning Optical Microscope

The NSOM successfully breaks the resolution limit of traditional optical microscope by detecting evanescent waves containing object details. Near-field scanning optical microscope has made significant advances in theory and practice, and is now being used in micron and nanotechnology. In a study published in 2002, RS Decca, Lee, and others proposed a system for tracking single molecules using a near-field scanning optical microscope (Decca et al., 2002). Meanwhile, AL Campillo’s group reported in 2001 plotting light intensity distribution in photonic crystals using a near-field scanning optical microscope (Campillo et al., 2001). In addition, progress has been achieved in the application of near-field scanning optical microscopes in biomedicine. CI Smith et al. (2018) used a quantum cascade laser in combination with an infrared aperture near-field scanning optical microscope to dramatically improve the capacity to identify esophageal cancer cells (Smith et al., 2018). It is believed that with the development of near-field optics, near-field optical microscopy will play a greater role in the micro- and nanotechnology.

#### 2.2.3 Application of Polarization Microscope

At present, polarized light microscope is playing a unique role in life science and material science. The polarization microscope is useful in biological studies because different biological tissues and even distinct proteins have variable polarization characteristics (He et al., 2021). In a 2016 study, J Chang et al. used a polarization microscope based on the DoFP polarimeter to examine living tissue samples (Chang et al., 2016). The results demonstrated that the DoFP polarimeter-based polarization microscope could monitor the dynamic process of biological samples in real time. It is reasonable to believe that this study will play an important role in the exploration of disease pathology in the future. At the same time, the polarization microscope also plays a unique role in material science. In 2019, Y Saito employed polarization Raman microscopy to assess the longitudinal strain of an epitaxial graphene monolayer on a Sic substrate, and the results revealed that polarization Raman microscopy could accurately assess the local stress of two-dimensional atomic materials (Saito et al., 2019).

#### 2.2.4 Application of Fluorescence Microscope

The fluorescent microscope is now employed in a variety of fields. Fluorescence microscopes are employed in biological molecular identification and genetic engineering in life science. Takao et al. examined the interior structure of a single DNA molecule employing optics under a fluorescent microscope in 2016. This research will be used to inform future DNA microstructure design (Takao et al., 2016). Fluorescence microscopy has also been used in material science at the same time. CAJ Putman et al. observed and imaged films LB using AFM and fluorescence microscopy in 2017 (Takao et al., 2016). The atomic force microscope and fluorescence microscopy are combined to consider the fluorescence microscope’s target localization and selection function as well as the atomic force microscope’s high-resolution imaging function. In the future, this combination will be used to create a new high-resolution imaging tool. For the examination of cell submicroscopic structure, super-resolution fluorescence microscopy is commonly used. In 2017, Matthew et al. employed super-resolution fluorescence microscopy to investigate the kinds and content of proteins during yeast division (Matthew et al., 2017).

#### 2.2.5 Application of Laser Confocal Scanning Microscope

Laser confocal scanning microscopes are now widely employed in sectors such as life sciences and materials science. Especially for life sciences, it has become an important research tool in the frontier research of life sciences. In a previous study, PJ Verschure applied laser confocal scanning microscopy to cartilage research in 1997. Their findings suggest that in the future, the application of laser confocal scanning microscopy will be important for studying the *in situ* immunolocalization of factors associated with joint pathology in intact cartilage (Verschure et al., 1997). In 2017, S Mursalimov’s team applies laser confocal scanning microscopy to analyze cell fusion studies in tobacco microspore cells. This study has provided the first 3D analysis of the cytological pattern of cell fusion in uncompressed cells. The results not only support the accuracy of results provided by other prior microscopy methods, but they also provide additional evidence that cell fusion is not caused by cell mechanics. This research will be useful for future cell fusion research (Mursalimov et al., 2019).

## 3 Electron Microscope

### 3.1 Transmission Electron Microscope

#### 3.1.1 Introduction to Transmission Electron Microscope

**FIGURE 5:**
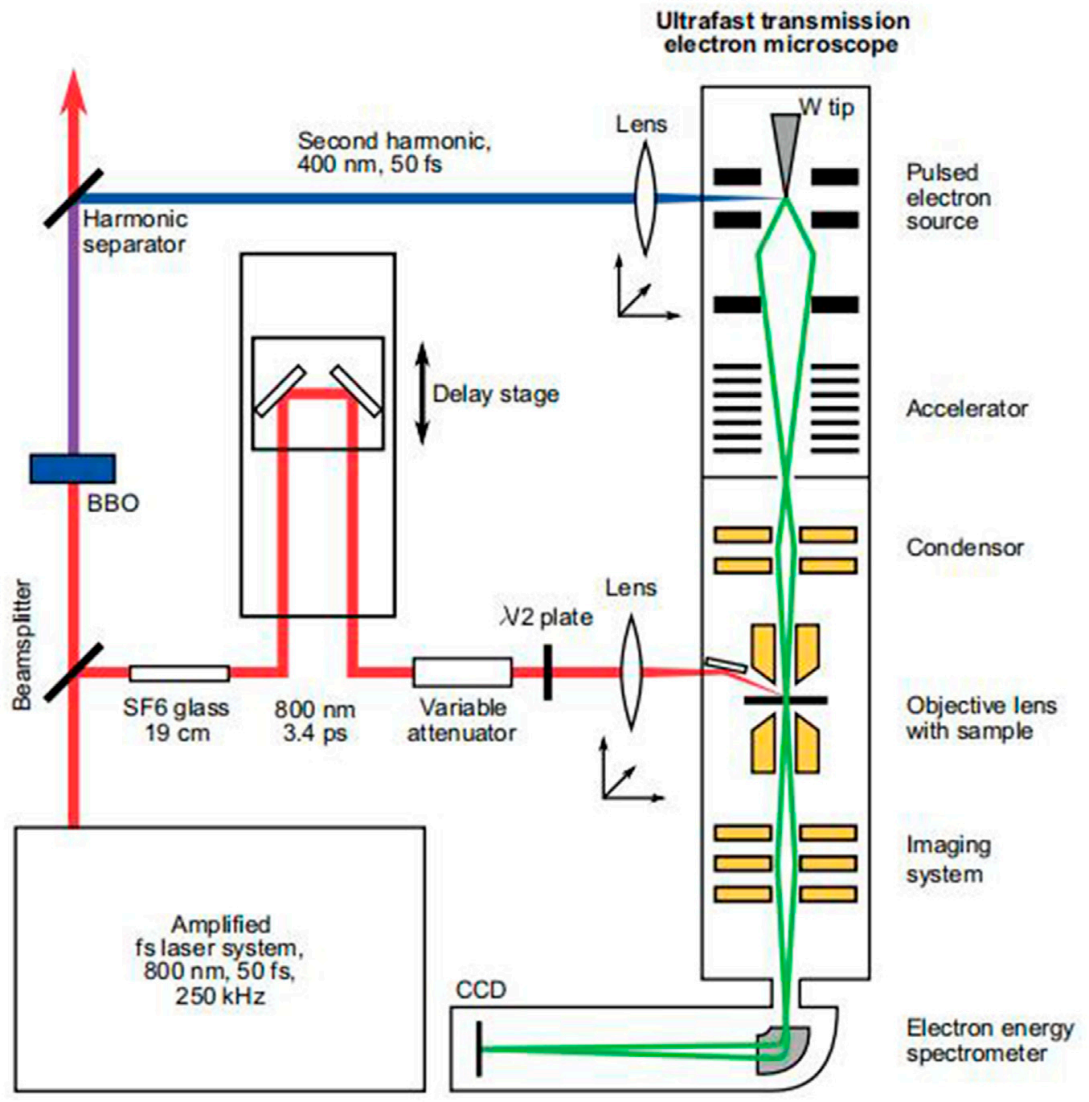
TEM principle diagram (Feist et al., 2015).

According to the calculation results of Abbe’s formula, the limit of the resolution of traditional optical microscopes is about 0.2 microns, which limits further exploration of the microscopic world by human beings. People began to recognize that electron microscope resolution might theoretically reach the atomic level as their understanding of electron characteristics improved, especially after the publication of de Broglie’s theory of matter waves in 1927. The interaction of electrons with solids is the fundamental premise of TEM. The TEM emits a beam of electrons to the sample under test while it operates in vacuum. Electrons scatter elastic and inelastic information as they move through the material. TEM can capture and extract information from dispersed electrons. In 2015, a Feist team published a work that included the TEM concept. The following illustration is taken from this study (Feist et al., 2015) to illustrate the TEM principle (Figure 5).

#### 3.1.2 Application of Transmission Electron Microscope

The most modern transmission electron microscopes now available have a spatial resolution of sub-angstroms, or less than 0.1 nm, with an energy resolution of more than 0.1 eV (Feist et al., 2015). The chemical composition and electronic structure of samples can also be determined using the energy spectrum, in addition to diffraction and imaging (Yang et al., 2014). Therefore, transmission electron microscopy is widely used in nanotechnology to observe the surface morphology, as well as to measure particle size and dispersion in the matrix. In a study, Christian et al. (2016) used a transmission electron microscope to tomography of cellulose nanocrystals aerogel, revealing the nanocrystalline aerogel of CNC′s detailed arrangement. This study will contribute to the further study of the properties of nanocrystal aerogel and its future applications (Buesch et al., 2016). Penetrating electron microscopy has been utilized to measure the physical properties of materials at the same time. In a study, P. Schattschneider et al. (2006) used transmission electron microscopy to identify magnetic circular dichroism. The combination of EMCD and TEM has been proposed as a critical microscopic approach for spintronics and nanomagnetism (Schattschneider et al., 2006). The TEM has been widely used in life science, particularly in the study of cell submicroscopic structures and biological macromolecules, as well as in the observation of life processes. In 2009, GB Chapman used transmission electron microscope TEM to observe the retina of zebrafish, and the results strongly support zebrafish as a vertebrate visual model, which is a useful tool for the study of vertebrates (Chapman et al., 2009). In 2018, J Abbas observed the antimalarial activity of bicolor calcification and heme crystal *in vitro* using TEM, which will contribute to the research and development of new antimalarial drugs (Abbas et al., 2018).

### 3.2 Scanning Electron Microscope

#### 3.2.1 Introduction to Scanning Electron Microscope

**FIGURE 6:**
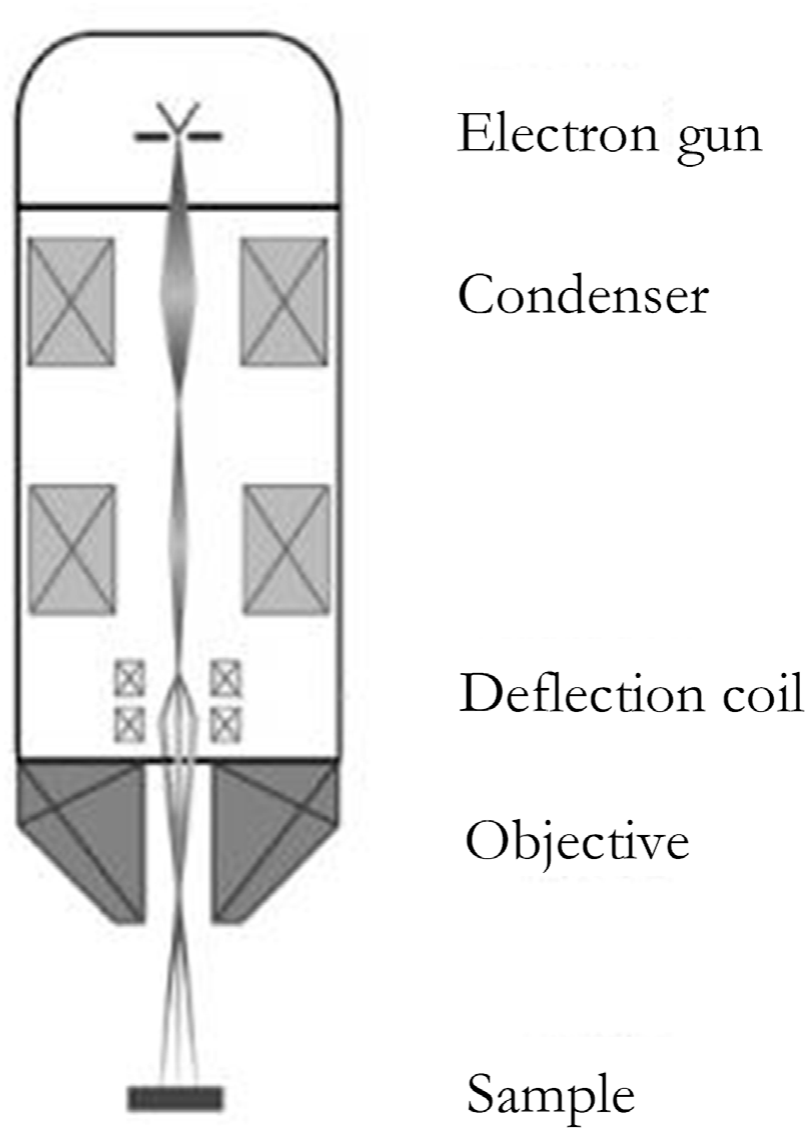
structure of the SEM (Chen et al., 2013).

**FIGURE 7:**
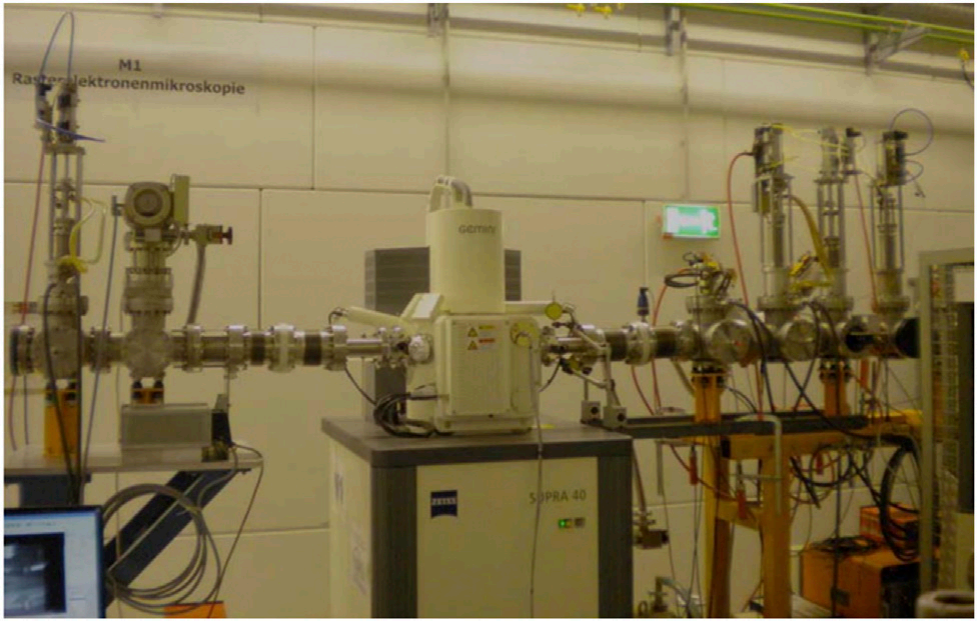
High-resolution scanning electron microscope (Amirthapandian, 2010).

With its ultrahigh resolution, the TEM, as the first invented electron microscope, has been widely employed. However, because scattering electrons are utilized to see things in TEM, there is some damage to the sample during observation, limiting its usage. The scanning electron microscope was created as the theory of electron microscopy progressed. A sample is scanned by a focused electron beam in scanning electron microscopy, which produces secondary or backscattered electrons as it contacts the material. SEM detects these electrons and converts them into pictures on the sample surface, which can be used to determine the particle size of nanoparticles (James et al., 2010). A secondary electron is a type of free electron created when an electron beam is used to bombard a sample, separating the atom’s outer electrons from the atom. The energy of the secondary electron is typically less than 50 eV. Secondary electron imaging (SEI) can analyze the sample surface with a high resolution of 1 nm since secondary electrons are created extremely close to the sample surface (usually 5–10 nm away from the surface) (Yan et al., 2018), as seen in Figure 6 (Chen et al., 2013) and Figure 7.

#### 3.2.2 Application of Scanning Electron Microscope

Advanced SEM measurement resolution can currently exceed 1 nm. In the realm of nanotechnology, SEM is used to assess the particle size of nanoparticles as well as their chemical composition. Simultaneously, scanning electron microscopy has been used in geological research to investigate the microstructure of rocks and soil, as well as the morphology and formation mechanism of crust. A work by Y Chen et al. (1980) used SEM to examine a soil sample. The findings demonstrate that observing crust morphology and development mechanisms using SEM on soil is beneficial. It has a significant impact on geology (Chen et al., 1980). JL Pilote studied the relative enrichment time of gold in volcanic massive sulfide deposits using scanning electron microscope mineral liberation analysis (SEM-MLA) in 2016, contributing to the research of deposit formation (Pilote et al., 2016). In the biomedical field, in addition to the traditional structure observation research direction, SEM has been applied in some research directions, which is emerging. In the biomedical field, RM Jarvis with Raman spectroscopy in a 2004 study interface of the scanning electron microscope (SEM) of bacterial surface-enhanced Raman spectra, which can identify the bacteria identification and characterization of a breakthrough in the study, laid the foundation for future single-cell level research (Jarvis et al., 2004). In a study, Zavialova et al. (2017) used scanning electron microscopy to reconstruct the ultrastructure of spore filaments, which will contribute to the structural analysis of fossilized macrospores (Zavialova and Karasev, 2017) and the combination of scanning electron microscopy and transmission electron microscope. Because SEM captures secondary electrons for image rendering, the imaging depth of field and visual field effect are good, allowing for clear images of the item to be measured using surface topography.

### 3.3 Scanning Transmission Electron Microscopy

#### 3.3.1 Introduction to Scanning Transmission Electron Microscopy

Crewe et al. (1968) pioneered field emission scanning transmission electron microscopy research, combining the advantages of the scanning electron microscope and transmission electron microscope, and using a field emission electron cannon as an electron source, considerably improving microscopic performance. In materials science and life science, scanning transmission electron microscopy has become a useful instrument.

#### 3.3.2 Application of Scanning Transmission Electron Microscopy

Because scanning transmission electron microscopy has a higher resolution, it has become an important experimental apparatus in the field of nanotechnology for information such as the structure of nanoparticles and the structural design of auxiliary nanomaterials. At the same time, scanning transmission electron microscopy is an important experiment instrument for the growth of nanocrystalline processes. In 2015, AV Levlev et al. used *in situ* liquid scanning transmission electron microscopy to study the growth process of platinum nanocrystals, which is important for optimizing nanocrystal preparation conditions and nanostructure synthesis (Ievlev et al., 2015). Masahiro Kawasaki et al. used scanning transmission electron microscopy to view graphite carbon nitride nanocomposite powder for a study in 2015, which will aid in the development of anode materials for high-energy lithium batteries (Kawasaki et al., 2015). On the other hand, scanning transmission electron microscopy is critical for microbial identification and observation of cell submicroscopic life activities. The microstructure of ferritin was studied using scanning transmission electron microscopy by N Jian et al. (2016). This research will aid in a better understanding of the iron nanoparticle formation mechanism in ferritin. Simultaneously, a medicine delivery system based on ferritin, as well as tailored treatment strategies for the disease (Jian et al., 2016).

## 4 Scanning Probe Microscope

### 4.1 Scanning Tunneling Microscope

#### 4.1.1 Introduction to Scanning Tunneling Microscope

**FIGURE 8:**
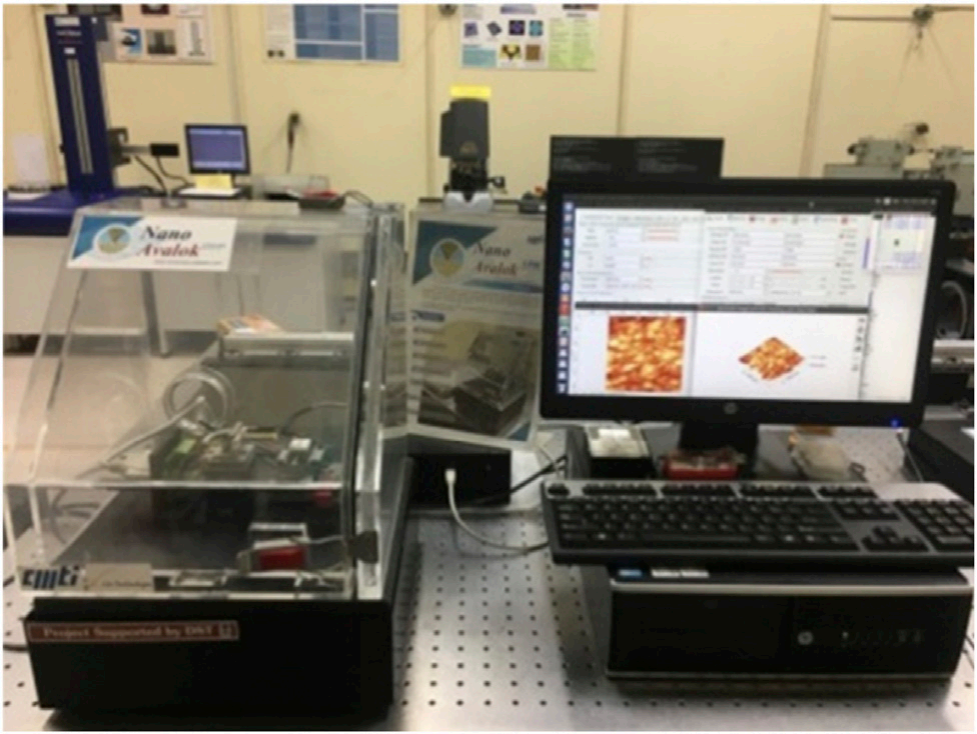
Scanning tunneling microscope (Snv et al., 2020)

With the continuous maturity and improvement of the theory of probe-principle microscopy, the scanning tunneling microscope was invented in 1980s. The probe is used by the STM to approach the surface of the item to be examined. At this time, due to its quantum mechanical properties, the electron will have the probability of quantum tunneling at the tip of the needle and the atomic surface constituting the object to be measured, and then produce electric current. The surface topography of the object can be examined by detecting the current. The invention of the scanning tunneling microscope is a milestone, giving humans the first opportunity to observe the arrangement of individual atoms on the surface of an object in real time. When the temperature falls below 4°C, the scanning tunneling microscope can be utilized as a processing tool to move individual atoms. Scanning tunneling microscopes are now widely employed in domains such as surface science, material science, and biological science. A laboratory scanning tunneling microscope image from SNV A research (Snv et al., 2020) is quoted here as a demonstration (Figure 8).

#### 4.1.2 Application of Scanning Tunneling Microscope

**FIGURE 9:**
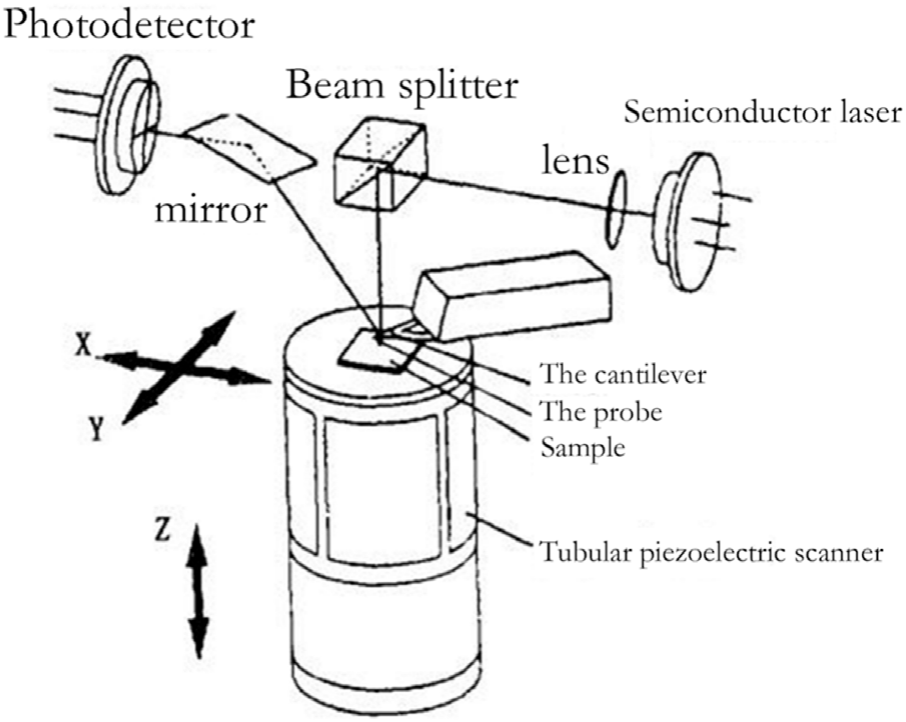
Schematic diagram of atomic force microscopy (Zhang, 2002).

Because it can directly detect the arrangement of atoms in the sample, the scanning tunneling microscope (STM) is currently widely employed in material research. At the same time, because the resolution of scanning tunneling microscopy reaches a single atom, it is employed as a detection method in nanotechnology to determine the particle size of nanoparticles. SNV A used a scanning tunneling microscope to observe gold nanoparticles made by the sputtering approach in 2020 and went on to conduct nanotechnology research (Snv et al., 2020). STM can also be utilized as a processing tool, which is particularly useful in nanotechnology. S Tjung et al. (2017) used scanning tunneling microscopy to carry out crystallization hydrogenation of graphene and studied the nanocrystalline structure produced by crystallization hydrogenation (Tjung et al., 2017), which will contribute to the processing and operation of scanning tunneling microscopy to process nanocrystals in the future. We have reason to anticipate that as scanning tunneling microscope (STM) applications continue to be researched, STM will become a key detection and processing tool for current promising emerging technologies such as nanotechnology (Figure 9).

### 4.2 Atomic Force Microscope

#### 4.2.1 Introduction to Atomic Force Microscope

**FIGURE 10:**
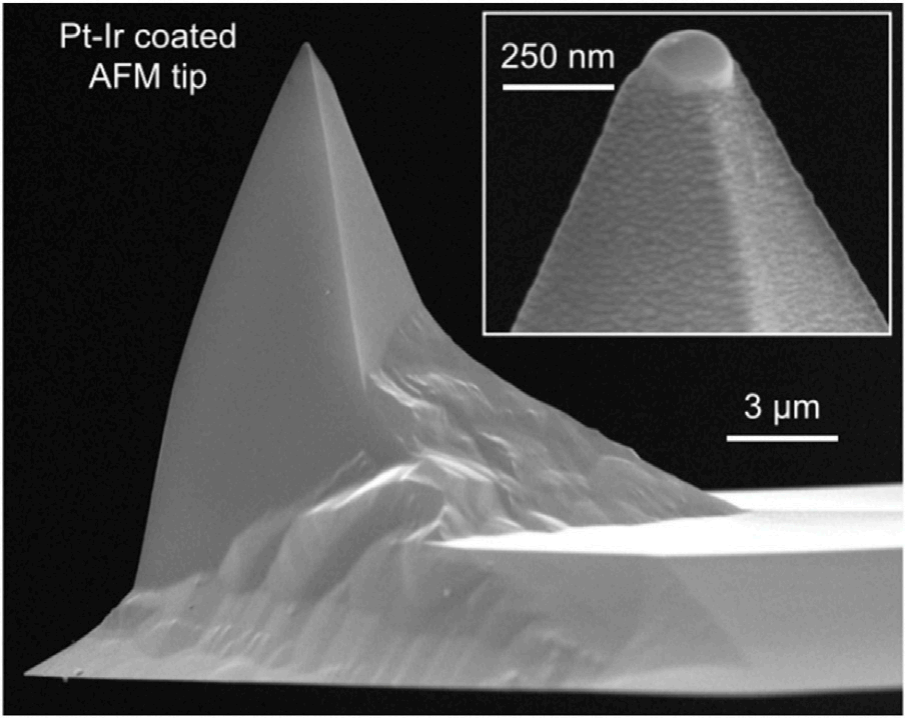
SEM image of AFM platinum-iridium layer probe (Kim et al., 2015).

With the invention of the scanning tunneling microscope, the probe microscope has become a new type of high-precision microscope. In 1986, G. Binning invented AFM using the STM probe measurement method. AFM currently has a bright future (Jie and Sun, 2005). The basic concept of AFM and the microstructure of an AFM probe under SEM observation are depicted in Figure 10.

Because the atomic force between atoms is a function of distance, measuring the atomic force of individual atoms can be used to determine the surface topography of samples. The atomic force microscope measures the atomic force on the sample surface through the probe. When the atomic force changes, the probe produces a small deformation relative to the initial state, which is amplified and transmitted to the position sensitive detector (PSD) through the optical lever, so as to convert the surface morphology signal of the sample into an electrical signal. Through continuous measurement, the morphology, particle size, chemical composition, and other characteristics of the sample to be tested are finally obtained.

#### 4.2.2 Application of Atomic Force Microscope

As one of the few tools that directly utilize atomic force, AFM has a resolution up to the atomic level, while the scanning tunneling microscope can arrange and process atoms at low temperatures. The atomic force microscope is now widely used in life science, material science, and other disciplines. The atomic force microscope (AFM) is an important tool in nanotechnology for detecting the particle size and chemical composition of nanoparticles as well as processing them. M Kim used an atomic force microscope to manipulate and assemble super-spherical AuNPs in 2015, resulting in supramolecules with uniform structure. Hyperspherical AuNPs’ dependable and deterministic operation will increase the accessibility of supramolecular bases and improve spatial positioning precision (Tjung et al., 2017). H Jin et al. (2016) used atomic force microscopy to detect the ultrastructure of erythrocytes of patients with type 2 diabetes and erythrocytes affected by aging in their research on type 2 diabetes, and found the difference in ultrastructure. This research will help us better understand the pathogenesis of type 2 diabetes from the ultrastructural level of red blood cells (Jin et al., 2016), and combined with this technology, it may be possible for forensics to use atomic force microscopy to judge the age of the deceased in the future.

## 5 Conclusion

From the perspective of development, this study systematically summarizes the development of microscopic imaging technology, and the principles, characteristics, and applications of various technologies in the field of micro-nanoscience as shown in the following table. The development of micro-imaging technology has greatly promoted the development of micro-nanoscience, and the human exploration demand in the field of micro-nanoscience also drives the development of micro-imaging technology. At present, the lower resolution limit of the traditional optical microscope is not enough to support the needs of micro-nanotechnology. The polarization microscope, fluorescence imaging microscope, and super-resolution microscope developed on the basis of the traditional optical microscope have brought new possibilities for further tapping the application potential of the optical microscope in micro-nanoscience. Although the resolution of an electron microscope is higher than that of a traditional optical microscope, its application environment must be vacuum. The resolution of the probe microscope reaches the atomic level, but the imaging speed is relatively slow. With the deepening of human understanding of the micro field, the requirements for micro-imaging technology will be higher and higher. Higher resolution, fewer environmental restrictions, and faster imaging speed will be the development direction of the new generation of the micro-imaging technology, which will be the breakthrough for the further development of micro-nanotechnology, and worth more energy from scientists, Table 1.

**TABLE 1:** Summary of the characteristics of all the microscopes mentioned in the study.

	Resolution	Work environment	Principle
Traditional optical microscope	0.2 µm	Atmospheric	Optical principle
Near-field scanning optical microscope	60–100 nm	Atmospheric	Near-field optics
Polarization microscope	0.2 µm	Atmospheric	Polarization optics
Fluorescence microscope	0.2 µm	Atmospheric	Fluorescence of the sample to be measured using UV excitation
Laser confocal scanning microscope	0.12 µm	Atmospheric	Laser scanning
Transmission electron microscope	0.2 nm	Vacuum	Collecting transmission electrons for imaging
Scanning electron microscope	1 nm	Vacuum	Collecting secondary electrons for imaging
Scanning transmission electron microscopy	1 nm	Vacuum	Collecting scattered electrons for imaging
Scanning tunneling microscope	0.2 nm	Vacuum	Exploiting the tunneling effect in quantum theory
Atomic force microscope	0.1 nm	Vacuum	Detecting changes in atomic forces on the surface of the object to be measured using a probe
