# Corrigendum: The development of microscopic imaging technology and its application in micro-and nanotechnology

**DOI:** 10.3389/fchem.2023.1351941

**Published:** 2023-12-14

**Authors:** Yong Wang, Xiushuo Zhang, Jing Xu, Xiangyu Sun, Xiaolong Zhao, Hongsheng Li, Yanping Liu, Jingjing Tian, Xiaorui Hao, Xiaofei Kong, Zhiwei Wang, Jie Yang, Yuqing Su

**Affiliations:** ^1^ Laboratory of Optical Detection and Imaging, School of Science, Qingdao University of Technology, Qingdao, China; ^2^ Quantum Physics Laboratory, School of Science, Qingdao University of Technology, Qingdao, China; ^3^ Qingdao Technology Innovation Center of Remote Sensing and Precise Measurement, Qingdao, China; ^4^ Torch High Technology Industry Development Center, Ministry of Science and Technology, Beijing, China

**Keywords:** microscopic, imaging, micro-nano, review, expectation

In the published article, there was an error in the **Funding** statement. One of the funders was missing. The correct **Funding** statement appears below:

“Open basic research project from the State Key Laboratory of Laser Interaction with Matter, China, No. SKLLIM2021‐11; Natural Science Foundation of Shandong Province, China, No. ZR2020QA078; National Natural Science Foundation of China, No. 12005110; Institute of Scientific and Technical Information of China, No. QN 2022-03.”

The authors apologize for this error and state that this does not change the scientific conclusions of the article in any way. The original article has been updated.

